# Development and validation of the tic score for early detection of traumatic coagulopathy upon hospital admission: a cohort study

**DOI:** 10.1186/s13054-024-04955-7

**Published:** 2024-05-18

**Authors:** Louis Brac, Albrice Levrat, Charles-Hervé Vacheron, Pierre Bouzat, Tristan Delory, Jean-Stéphane David

**Affiliations:** 1 Department of Intensive Care, Annecy-Genevois Hospital, Annecy, France; 2 Department of Anesthesia and Intensive Care, Groupe Hospitalier Sud, Hospices Civils de Lyon, Pierre Bénite, France; 3 Biostatistics Health Team, Biometrics and Evolutionary Biology Laboratory, Hospices Civils de Lyon, Lyon, France; 4 Department of Anesthesia and Intensive Care, Grenoble-Alpes University Hospital, Grenoble, France; 5 Annecy-Genevois Hospital, Annecy, France; 6 INSERM, Institut Pierre Louis d’Épidémiologie et de Santé Publique, Sorbonne Université, Paris, France; 7 Department of Anesthesia and Intensive Care, Lyon Sud Hospital, Hospices Civils de Lyon, Pierre-Bénite, France; 8 Research on Healthcare Performance (RESHAPE), INSERM U1290, University Claude Bernard Lyon 1, Lyon, France

**Keywords:** Coagulopathy, Score, Trauma, Prehospital, Prediction, Blood products

## Abstract

**Background:**

Critically injured patients need rapid and appropriate hemostatic treatment, which requires prompt identification of trauma-induced coagulopathy (TIC) upon hospital admission. We developed and validated the performance of a clinical score based on prehospital resuscitation parameters and vital signs at hospital admission for early diagnosis of TIC.

**Methods:**

The score was derived from a level-1 trauma center registry (training set). It was then validated on data from two other level-1 trauma centers: first on a trauma registry (retrospective validation set), and then on a prospective cohort (prospective validation set). TIC was defined as a PT_ratio_ > 1.2 at hospital admission. Prehospital (vital signs and resuscitation care) and admission data (vital signs and laboratory parameters) were collected. We considered parameters independently associated with TIC in the score (binomial logistic regression). We estimated the score’s performance for the prediction of TIC.

**Results:**

A total of 3489 patients were included, and among these a TIC was observed in 22% (95% CI 21–24%) of cases. Five criteria were identified and included in the TIC Score: Glasgow coma scale < 9, Shock Index > 0.9, hemoglobin < 11 g.dL^−1^, prehospital fluid volume > 1000 ml, and prehospital use of norepinephrine (yes/no). The score, ranging from 0 and 9 points, had good performance for the identification of TIC (AUC: 0.82, 95% CI: 0.81–0.84) without differences between the three sets used. A score value < 2 had a negative predictive value of 93% and was selected to rule-out TIC. Conversely, a score value ≥ 6 had a positive predictive value of 92% and was selected to indicate TIC.

**Conclusion:**

The TIC Score is quick and easy to calculate and can accurately identify patients with TIC upon hospital admission.

**Supplementary Information:**

The online version contains supplementary material available at 10.1186/s13054-024-04955-7.

## Introduction

Severe injuries are a leading cause of death globally, particularly among young people [1]. Uncontrolled bleeding and traumatic brain injury are the primary causes of death in these patients, and trauma-induced coagulopathy (TIC) is frequent in such cases (up to a third of patients) [2]. TIC is associated with impaired outcomes, including an increased likelihood of massive bleeding, multiple organ failure, transfusion, and death in the first hours following hospital admission [3–5]. Early and aggressive resuscitation strategies that aim to directly correct TIC are associated with improved outcomes and decreased blood products administration [6, 7], and therefore the early identification and prompt treatment of TIC are essential [8]. However, in daily practice, the administration of hemostatic resuscitation is often based on clinical judgment, which performs moderately to identify massive hemorrhage [9], leading to unnecessary blood product administration to patients responsible for potential side effects and wasted blood products [10].

TIC is most commonly defined as a PT_ratio_ measurement > 1.2 [11]. It is usually measured using conventional coagulation techniques or point-of-care devices [12, 13], including viscoelastic techniques (VET) [14], but they have several limitations and are not available in all hospitals. In addition to point-of-care devices, it has been also suggested to calculate scores upon patient admission for predicting TIC or the need for massive transfusion [15–17]. These scores usually require additional laboratory data such as arterial blood gas measurement, a FAST (focused assessment with sonography in trauma) or a full clinical examination. Perkins et al. have proposed a score based on 14 variables that must be calculated online, and which is strongly associated with TIC [16]. Two other scores, COAST and PACT, have also been proposed [15, 18]. Both can be calculated before hospital admission. The performance of the COAST score is weak as sensitivity is poor; that of the PACT score is better, but it only considers certain elements of pre-hospital resuscitation, such as cardiopulmonary resuscitation or intubation and it requires an online application for calculation [15]. In a previous study, we observed that several parameters, including pre-hospital vital signs (Glasgow coma scale and Shock Index) pre-hospital resuscitation (fluids and vasopressors), as well as parameters measured at admission (Shock Index and point-of-care hemoglobin) were associated with TIC upon admission [19].

The aim of the present study was to create and validate a straightforward screening tool, called the *Trauma Induced Coagulopathy* (TIC) Score, to rapidly identify patients with a PT_ratio_ > 1.2.

## Material and methods

### Study design and data collection

To build the score, we used data from the registries of three regional trauma centers (Lyon-Sud university hospital, Grenoble university hospital, and Annecy Genevois hospital). We retrieved the demographic and injury characteristics for each patient, including the Injury Severity Score (ISS), prehospital and admission vital signs, prehospital resuscitation including fluids volume, tranexamic acid and vasopressor, and survival at hospital discharge. Point-of-care hemoglobin (HemoCue® France, Bailly-Romainvilliers, France) was measured at admission. TIC was defined as a PT_ratio_ > 1.2 [2, 3]. We excluded patients who were treated with anticoagulants, received fresh frozen plasma or platelet concentrates during the prehospital phase. Additionally, we excluded patients without hemostasis analysis at admission. The study was approved by the ethics committee of the *French Society of Anesthesia and Intensive Care* (00010254-2021-217), and it was registered under the number (*Commission Nationale Informatique et Liberté*, MR004-2205982). Information about the registry was provided to all patients (or their next of kin), and written informed consent was not required. This study follows the TRIPOD Statement for prediction model studies [20].

The score was derived from the first set of data (training set) obtained from the trauma registry in Lyon. We then validated the predictive values of the TIC Score retrospectively on a trauma registry (retrospective validation set) common to Grenoble and Annecy trauma centers. Finally, we validated the performance of the TIC Score, in a third set, on data prospectively collected in two trauma centers (Grenoble university hospital and Genevois-Annecy hospital), that were required for computing individual score value.

### Patient care

In France, all patients are cared for and triaged during the prehospital phase by a physician who may be an anesthesiologist or an emergency medicine physician (‘SAMU system’) [21, 22]. After careful evaluation of injury severity (clinical examination and vital signs, FAST examination, point-of-care hemoglobin), the prehospital physician implements all the necessary care including resuscitation techniques (mechanical ventilation, blood transfusion, general anesthesia, vasopressor and fluid resuscitation, analgesia, etc.) and direct the patient to the most appropriate facility. Fluid resuscitation usually includes crystalloids such as saline or a balanced solution (Ringer's lactate). Administration of norepinephrine is suggested if, despite fluid resuscitation, systolic blood pressure remains below 80–90 mmHg or in the case of severe head trauma, if systolic blood pressure remains below 110–120 mmHg. The three trauma centers have similar practices in the anesthesiologist-led trauma resuscitation unit. This includes blood testing strategy and algorithmic-based decision-making process for delivering blood products. A viscoelastic assay is used to diagnose a TIC and to guide administration of blood products in the three trauma centers.

### The TIC score

#### Training set

We developed the score using data from a training cohort retrieved from a prospective registry supervised by the regional emergency network ‘RESUVAL’. We included from the registry severely injured patients admitted to the Lyon Sud university hospital, a level-1 trauma center, between January 1, 2011, and December 31, 2019, who met at least one of the following criteria: (1) received at least one blood product or coagulation factor concentrate during the first 24 h following hospital admission; (2) had a ROTEM (Werfen, le Pré St Gervais, France) or conventional laboratory tests to measure hemostasis; (3) were admitted to a critical care unit.

Factors independently associated with a PT_ratio_ > 1.2 at admission were identified, using bi-directional stepwise logistic binomial regression, among the following: sex, type of injury (factor, blunt versus penetrating), Glasgow coma scale at first medical evaluation (numeric value, between 3 and 15), prehospital and admission systolic blood pressure (numeric value, in mmHg), heart rate (numeric value, in bpm) and Shock index (numeric value, heart rate/systolic blood pressure), admission point-of-care hemoglobin level (numeric value, in g.dL^−1^), prehospital resuscitation using fluid therapy (factor, yes/no), and prehospital use of norepinephrine (factor, yes/no). Each numeric variable was categorized using the median, or the most relevant cut-off. Associations were reported as odds ratio (OR) with 95% confidence intervals (CI). We derived the score form from the final model’s coefficients (β) of the factors that were significantly associated with the outcome in the regression analysis, using a previously described methodology [23].

#### Retrospective validation set

To ensure the generalizability of the score, we first validated the TIC Score using data from a regional trauma registry in which data are prospectively collected, including all adult patients admitted with a severe injury according to regional triage rules, and admitted to one of two level-1 trauma centers (Grenoble university hospital, and Annecy Genevois hospital, France—the ‘TRENAU’ registry) between January 1, 2018 and December 31, 2020 [22].

#### Prospective validation set

To control for the level of missingness for the variables of interest and optimize methodological reliability, we constituted a prospective validation cohort of all consecutive patients admitted with severe injury to the same two trauma centers (Grenoble university hospital, and Annecy Genevois hospital, France—the ‘TRENAU’ registry) between February 1 and May 31, 2022. Data collected in this cohort were crosschecked by independent examiners.

### Statistical analysis

We used median and interquartile range [IQR] to describe numeric variables, and number and percentage (%) for nominal variables. Differences between groups were estimated using the Wilcoxon rank-sum test for quantitative variables, and the Chi-squared test or Fisher’s exact test for qualitative variables.

#### Score performances

For each of these datasets, we calculated the performance of the score to predict a coagulopathy (PT_ratio_ > 1.2), including sensitivity and specificity, negative and positive predictive values, as well as the discrimination (receiver operating characteristic curve) and the calibration (calibration plot and Brier score). We also calculated the predicted probability of coagulopathy associated with each score value.

As INR is used by some in place of PT_ratio_, we also checked that substituting INR for PT_ratio_ did not alter the results; in addition the performance of the TIC Score to predict fibrinogen < 1.5 g.L^−1^.

For the training set and the first validation set, we used *Multivariate Imputation by Chained Equations* (MICE) to handle missing information. In the second validation set, we did not impute for missing data. We performed a sensitivity analysis by estimating the performance of the score on the merged datasets including all patients with complete data. All tests were two-tailed, and significance set at 5%. All analyses were performed using the R software for statistical computing, version 3.4.3 (R foundation for statistical computing, Vienna, Austria).

## Results

**Fig. 1:**
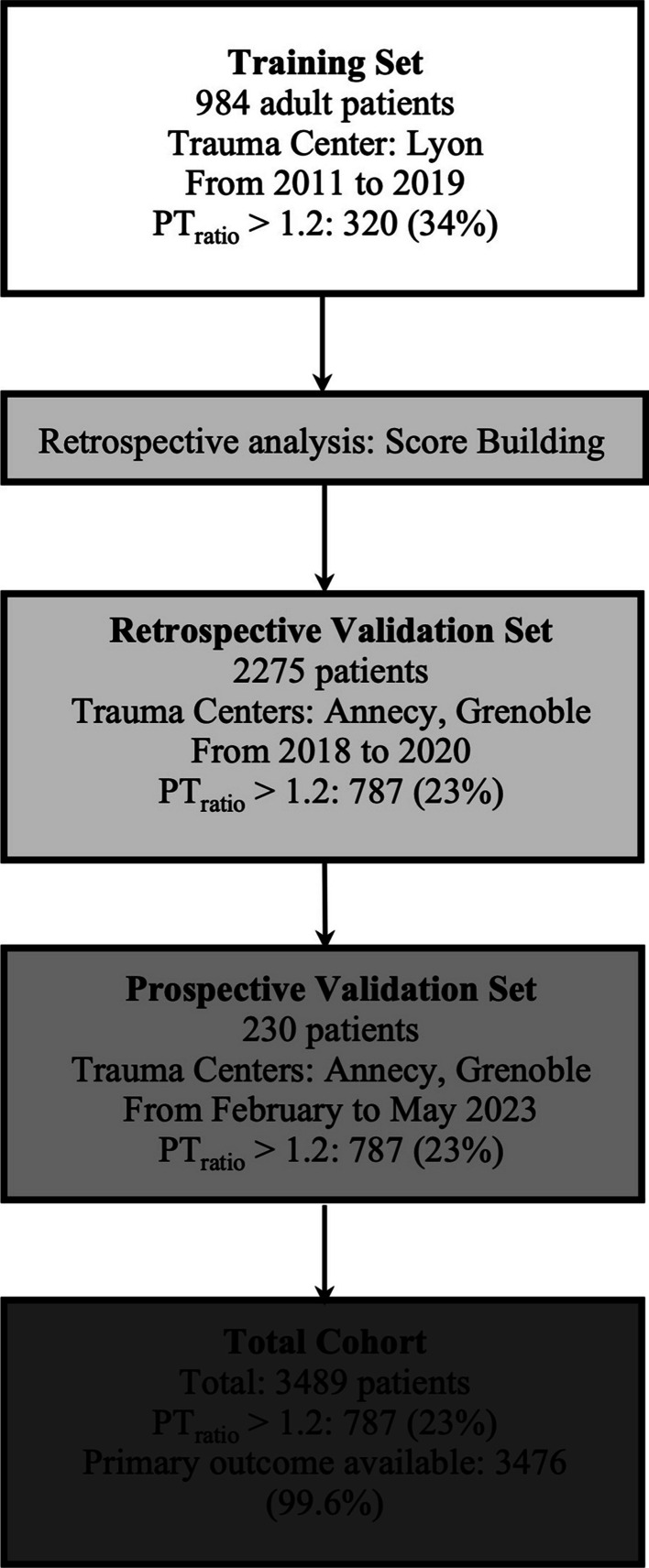
Flowchart of the study. *Exclusion criteria*: Patients < 18 years old, receiving anticoagulant therapy, prehospital administration of fresh frozen plasma/fibrinogen concentrate

In total, 3,489 trauma patients were included in the study: 984 (28%) in the training set, 2275 (65%) in the historical validation set, and 230 (7%) in the prospective validation set (Fig. 1). Overall, the rate of TIC was 22% (95% CI 21–24%). Patients were severely injured (median [IQR] ISS 17 [9–26]), and their injury was overwhelmingly blunt trauma (93%, Table 1). The characteristics of each set are described in Table 1.

**Table 1:** Baseline demographics and vital signs. Data are median [interquartile range] or n (%)

Characteristics	Training Set	Retrospective validation set	Prospective validation set	Data available
*N*	984	2275	230	3489
*Demographics and injury characteristics*				
Age	41 [25–57]	40 [28–56]	37 [25–56]	3455 (99)
Male sex	748 (76)	1805 (79)	164 (73)	3489 (100)
Penetrating injury	62 (6)	169 (7)	13 (6)	3489 (100)
ISS	25 [17–33]	13 [9–25]	16 [9–25]	3484 (100)
ISS *categories*				
< *16*	172 (18)	1340 (59)	109 (47)	
*16–24*	306 (31)	377 (17)	66 (29)	
*25–48*	445 (45)	526 (23)	44 (19)	
*49–75*	61 (6)	28 (1)	19 (8)	
*Prehospital characteristics*				
Shock Index > 0.9	245 (25)	300 (18)	40 (18)	3274 (94)
GCS < 9	290 (30)	326 (15)	34 (15)	3429 (98)
TXA administration	497 (52)	528 (37)	149 (66)	2625 (75)
Norepinephrine use	201 (20)	111 (5)	34 (15)	3488 (100)
Prehospital RBC	41 (4)	13 (1)	4 (2)	3489 (100)
*Admission characteristics*				
Shock index, *continuous*	0.7 [0.6–0.9]	0.7 [0.6–0.8]	0.7 [0.6–0.8]	3274 (94)
Shock Index > 0.9	295 (25)	295 (13)	40 (18)	3274 (94)
*Laboratory tests*				
POC Hgb	125 [108–139]	139 [129–151]	137 [135–150]	3444 (99)
POC Hgb < 11 (g.dL^−1^)	252 (26)	237 (10)	36 (16)	3444 (99)
PT_ratio_	1.1 [1.0–1.3]	1.1 [1.0–1.2]	1.1 [1.0–1.2]	3476 (100)
PT_ratio_ > 1.2	320 (34)	412 (18)	51 (22)	3476 (100)
Hemoglobin (g.dL^−1^)	126 [110–140]	139 [129–151]	137 [123–149]	3460 (99)
Fibrinogen (g.L^−1^)	2.1 [1.7–2.6]	2.7 [2.2–3.2]	2.5 [2.1–2.9]	3370 (96)
Platelet (10^9^.L^−1^)	213 [179–259]	238 [200–279]	241 [198–290]	3453 (99)

Data are median [interquartile range] or n (%)

*GCS* Glasgow coma scale; *TXA* tranexamic acid; *RBC* red blood cell; *POC* point-of-care; *PT*_*ratio*_ prothrombin time ratio; *Hgb* hemoglobin

### The TIC score

From regression analysis, and among the 984 patients in the training set, factors associated with a coagulopathy upon trauma center admission were: prehospital GCS < 9; admission point-of-care hemoglobin level < 11 g/dL; admission Shock Index value > 0.9; prehospital fluid therapy above 1000 ml; and prehospital norepinephrine (Table 2). These variables were included in the computation of the TIC Score, for which values ranged from 0 to 9. The score coefficients and score points are presented in Table 2.

**Table 2:** Factors associated with coagulopathy, and variables retained in the score in multivariate regression analysis

TIC Score variables	Coefficient	Odds ratio (95% CI)	Corresponding Score points
Point-of-care hemoglobin level < 11 (g.dL^−1^)	1.47	4.37 (3.06–6.23)	3
Shock Index > 0.9	1.13	3.10 (2.19–4.40)	2
GCS < 9	0.98	2.67 (1.87–3.82)	2
Prehospital Fluid resuscitation > 1000 ml	0.82	2.24 (1.16–2.68)	1
Prehospital norepinephrine	0.56	2.27 (1.60–3.23)	1
Total			9

Intercept of construction model: −2.30 [−2.57 to −2.04], *p* < 0.001

**Fig. 2:**
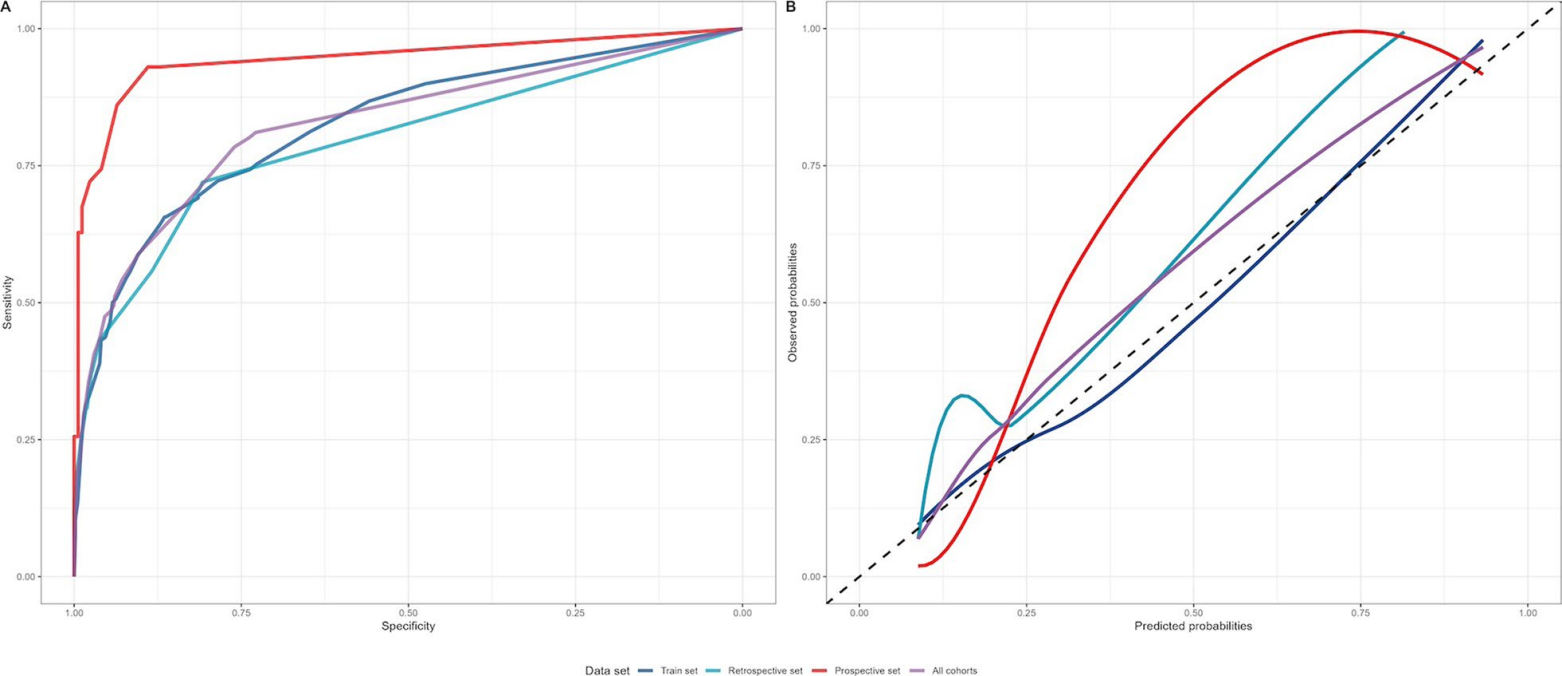
TIC Score receiving operating curve and calibration plot for predicting PT_ratio_ > 1.2, by dataset and overall. Panel **A**: ROC curve. Plot of the TIC Score sensitivity by specificity for predicting PT_ratio_ > 1.2, by dataset; Panel **B**: Calibration Plot. Plot of observed probabilities by the probabilities predicted by the TIC Score for observing a PT_ratio_ > 1.2, and dataset. Lines are smoothed using coefficients from linear regression: blue line indicates Training Set, Sky blue line indicates retrospective Set, red line indicates prospective set and purple line indicates All Cohorts

We defined a score value < 2 as the threshold for a low probability of coagulopathy, and a score value ≥ 6 as the one for a high probability of coagulopathy. Score values between 2 and 5 indicate possible coagulopathy (Table 3). Among the 3075 patients with complete data across different datasets, 1967 (64%) had a score < 2900 (29%) had a score between 2 and 5, and 208 (7%) had a score ≥ 6. Fibrinogen, PT_ratio_ and platelet count by score category, are presented in Supplementary Fig. S1. Across all datasets, the score yielded an area under the curve (AUC) of 0.82 (95% CI 0.81–0.84) to predict TIC (Fig. 2A). The calibration was 0.115 according to the Brier score, without significant differences across sets (Fig. 2B). Details of the performance and calibration of the TIC Score on the training and validation sets are presented in Table 3.

**Table 3:** Performance and calibration of the TIC score on training and validation sets

Score	Training set			Validation set			Prospective set			Total population		
	Probability	PPV	NPV	Probability	PPV	NPV	Probability	PPV	NPV	Probability	PPV	NPV
0	9.1	44.7	90.4	7.3	45.1	93.1	2.9	64.5	98.0	7.8	45.9	93.1
1	14.7	49	89.4	15.0	45.7	92.7	9.4	67.8	98.1	14.2	48.2	92.5
2	23.0	58.4	85.1	28.3	70.0	88.7	26.7	82.1	93.8	24.4	64.2	88.4
3	33.0	70.5	83.5	46.9	78.2	86.9	56.1	96.4	91.4	38.8	74.8	86.5
4	47.0	76.1	81.1	66.4	84.3	85.8	81.7	95.8	89.5	55.3	79.8	85.1
5	60.5	82.4	77.5	81.5	96.3	83.8	94.0	94.4	86.8	70.8	86.5	82.5
6	72.5	89.1	74.6	90.8	97.4	83.2	98.2	92.3	84.7	82.6	92.0	81.1
7	82.0	91.7	70.5	95.7	100	82.5	99.5	100	83.5	90.3	94.9	79.3
8	88.7	96.2	69.2	98.0	100	82.0	99.9	100	81.9	94.8	96.7	78.6
9	93.1	100	67.5	99.1	100	–	100	100	80.0	93.3	100	77.9
AUC (95% CI)	0.82 (0.79–0.85)			0.80 (0.77–0.82)			0.93 (0.89–0.96)			0.83 (0.81–0.84)		

*PPV* positive predictive value; *NPV* negative predictive value. Calibration for total population (Brier: 0.115). AUC (area under the curve); 95% CI: 95% confidence interval

We explored if the INR could be used to define TIC instead of PT_ratio_ to establish the scoring system. The AUC of the score to predict a TIC defined as an INR > 1.2 instead of PT_ratio_ > 1.2, was 0.80 (95% CI 0.79–0.82), which was not significantly different from the AUC calculated with the PT_ratio_ (*p* = 0.097).

We also observed that the TIC Score well predicted fibrinogen < 1.5 g.L^−1^ (AUC: 0.88, 95% CI: 0.86 to 0.90) and a threshold ≥ 6 had a 97% specificity (Supplementary Fig.  S2).

### Outcomes and blood products administration

**Fig. 3:**
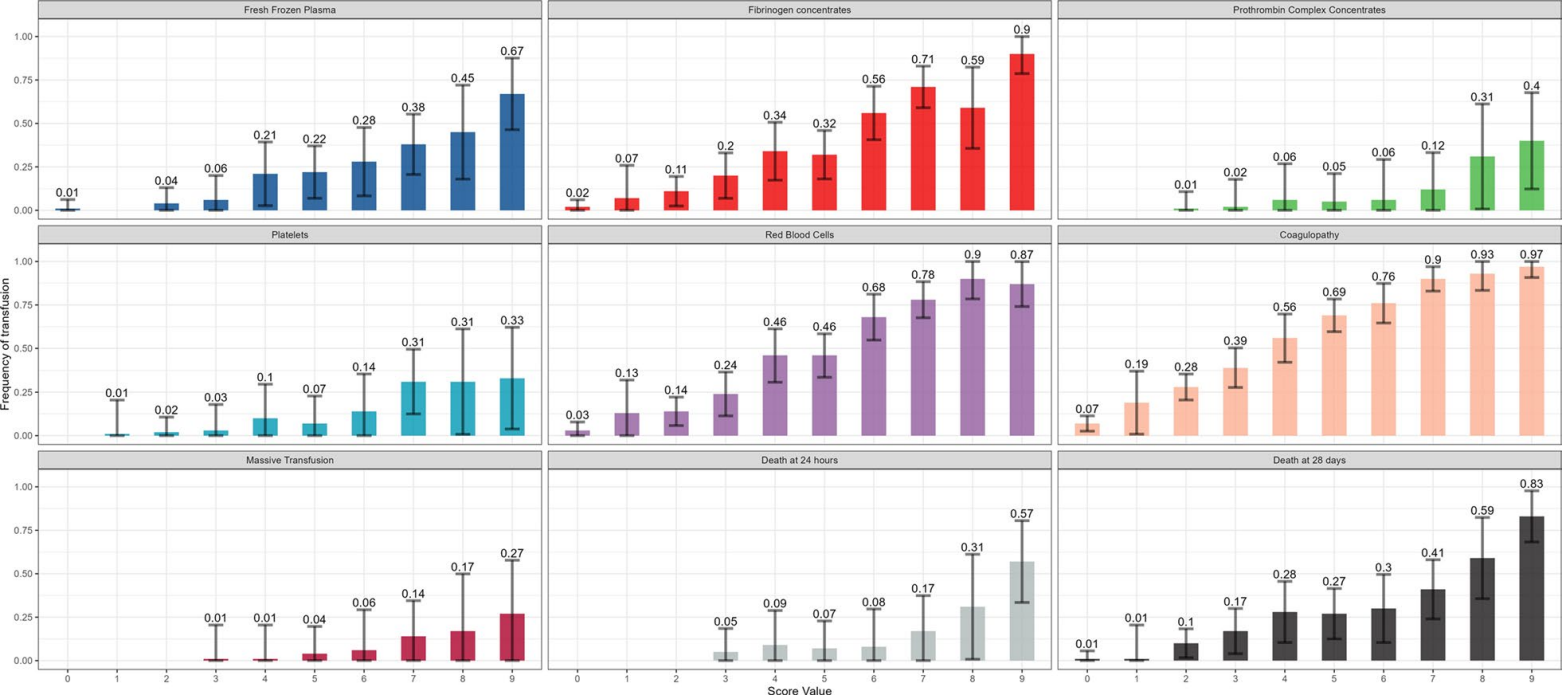
Outcome according to score values. Bars correspond to frequency of event (percentage) by score value (ranging from 0 to 9). The 95% CI are displayed for each point using error bars in lightgrey

Overall, mortality was 10.0% (95% CI: 8.9% to 11.1%) at hospital discharge. We observed a close relationship between the TIC Score and the administration of blood products, massive transfusion rate, mortality at 24 h and at hospital discharge (Fig. 3).

## Discussion

We have developed a simple and ready-to-use screening tool for the early detection of TIC. The TIC Score has good statistical performance; a TIC Score < 2 indicating patients with a low probability of TIC, and a TIC Score ≥ 6 identifying patients with a high probability of TIC.

Trauma patients require hemostatic goal-directed therapy that relies on early and accurate diagnosis of TIC to provide optimal blood support [6, 8, 14, 24]. Laboratory tests used to guide hemostatic therapy can be either conventional coagulation tests or viscoelastic assays [8]. Viscoelastic assays allow timely management of trauma by providing a 30–60-min gain over conventional coagulation tests [14]. A randomized controlled trial of patients requiring massive transfusion showed improved survival at 28 days for those allocated to receive a massive transfusion protocol guided by viscoelastic testing compared with conventional coagulation testing [6]. However, these results were not replicated in the ITACTIC randomized controlled trial [25], probably due to a bias towards a population at very low risk of trauma-induced coagulopathy [26]. These data highlight the need for a screening tool able to accurately detect the presence of a TIC upon patient admission. The TIC Score will allow the selection of patients with a high probability of trauma-induced coagulopathy, i.e. those who could benefit the most from early initiation of active therapies, including fibrinogen (TIC Score ≥ 6). It is for these patients that the use of VETs could be most interesting and relevant. Conversely, for patients with a low probability of coagulopathy (TIC Score < 2, 64% of cases) the administration of blood products will be avoided and also the many associated side effects (TRALI, infection, venous thrombosis, etc.).

Other scores have been developed for the prediction of massive blood transfusion (ABC, TASH, TICCS), or to assess the presence of a coagulopathy (PACT, TICCS, COAST) [16, 17, 27–29]. However, although these scores can predict massive transfusion or coagulopathy, they were not developed to implement a goal-directed therapy algorithm [29, 30]. Only the Bayesian score has been specifically developed to identify a PT_ratio_ > 1.2, with similar performance to the TIC Score described herein [31], but it is not suitable for the early management of severely injured patients because of its complexity (14 variables including 3 laboratory variables) that precludes its timely calculation at the admission to the trauma center. Other scores have a low sensitivity for predicting coagulopathy (21% for TICCS and 17% for COAST), resulting in a high likelihood of false negatives and in the risk of missing opportunities for early detection and correction of TIC [29, 32]. It has been emphasized that in the absence of external and prospective validation of these scores, their reliability is too low to promote their use in daily practice [30, 33].

While PT_ratio_ and INR values may not be directly interchangeable due to variations in thromboplastin sensitivity, the consistent performance of the TIC Score regardless of the parameter used suggests its robustness in characterizing coagulopathy. This flexibility enhances the applicability and usability of the TIC Score across different healthcare settings and practices.

**Fig. 4:**
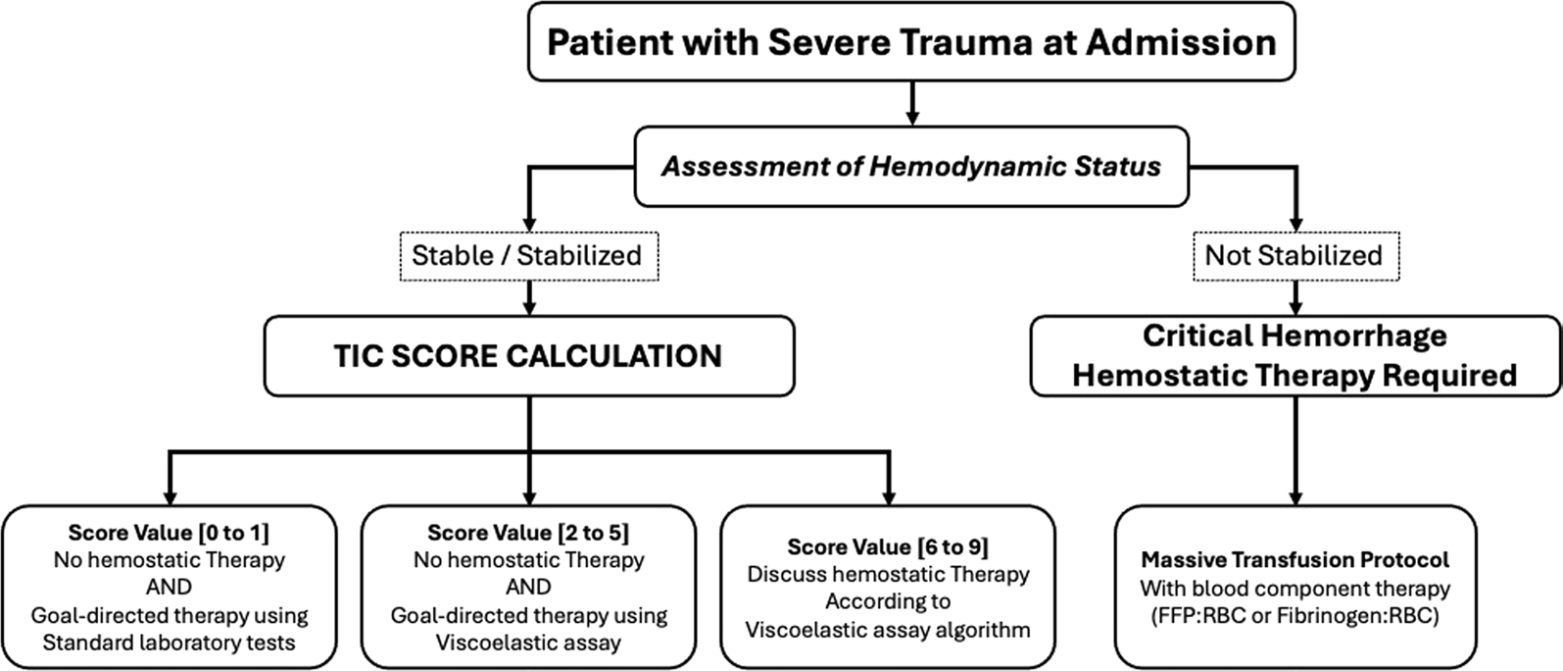
Suggested triage algorithm using the TIC Score. This algorithm proposes à patient management strategy found on clinical features and TIC Score values. *FFP* fresh frozen plasma; *RBC* red blood cell

Finally, we propose a simple triage algorithm (Fig. 4) that strikes a balance between sensitivity and specificity, and could enable, in a straightforward manner, a more targeted and specific use of VETs and, where appropriate, blood products.

### Study strengths

The multicenter design in highly experienced level-1 trauma centers, with a large sample size mixing data from trauma registries with prospective evaluations, supports the robustness and reliability of interpretation. The TIC Score is operational and pragmatic, with variables systematically and immediately available upon admission to the trauma center. Several key factors justify the extrapolation of the results across centers: the wide range of trauma types and severity of injury as well as the various geographical areas (rural, mountainous and urban). Finally, we also observed that INR can be used as an alternative to the PT_ratio_, thus increasing the possibilities of using the TIC Score.

### Study limitations

The registry data used to develop the TIC Score had missingness (~ 12%). However, in the prospective dataset the level of missingness was very low, and the performance of the score remained similar. The performance of the score was also consistent in the analysis including all patients with complete data. However, patients presented mainly with blunt trauma, and only 7% with penetrating trauma, as is usually the case in France [34, 35]. This is well below that observed in American trauma centers, where ballistic trauma is more commonly observed [34]. Another limitation is that the study was conducted in a trauma system where patients are cared by a physician and then received extensive prehospital resuscitation, including fluid resuscitation and vasopressors, intubation and mechanical ventilation, chest tube, and, if available, blood products. The results presented herein may therefore not be transposable to systems where patients do not benefit from intensive prehospital resuscitation and where priority is given to rapid transport. A final limitation may arise from the fact that norepinephrine administration and the Shock Index co-exist in the score, and that there may be interactions between the 2. This is a parameter to be taken into account, but several years ago we showed that the Shock Index measured during the patient's initial pre-hospital assessment was associated in multivariate analysis with the occurrence of coagulopathy or massive transfusion, after adjustment on several parameters including norepinephrine administration [19].

## Conclusion

The TIC Score is an easy to calculate score for the early diagnosis of TIC at admission. It may help for the selection of patients in need of viscoelastic testing or a hemostatic treatment. A score < 2 rules out a TIC whereas a score ≥ 6 indicates a TIC.

## Supplementary Information

Supplementary file 1 (JPG 1471 kb)

Supplementary file 2 (JPG 453 kb)

Supplementary file 3 (DOCX 12 kb)

## Data Availability

On demand to the authors.
