# Supplementary figures and images for: Development and validation of the tic score for early detection of traumatic coagulopathy upon hospital admission: a cohort study
Source: Crit Care. 2024 May 18;28:168. doi: 10.1186/s13054-024-04955-7 (PMC11102139; doi:10.1186/s13054-024-04955-7)

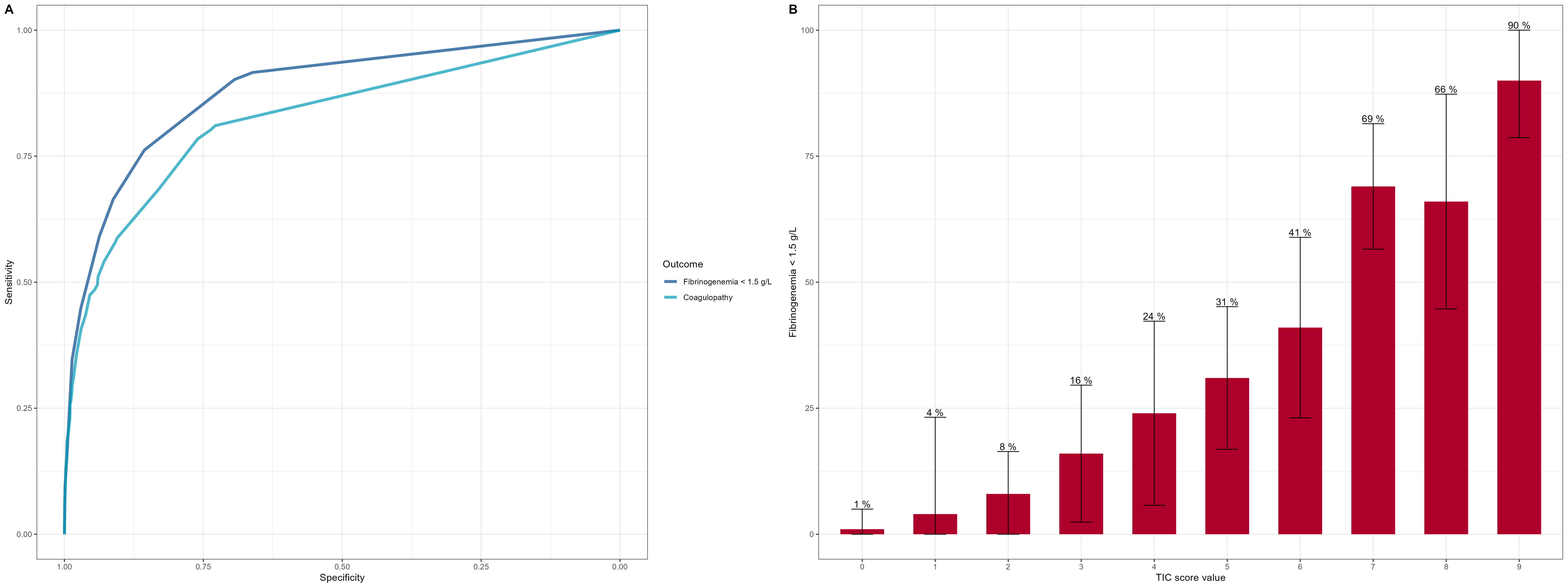

Supplement: Supplementary file 1 — (JPG 1471 kb) [file 13054_2024_4955_MOESM1_ESM.jpg]

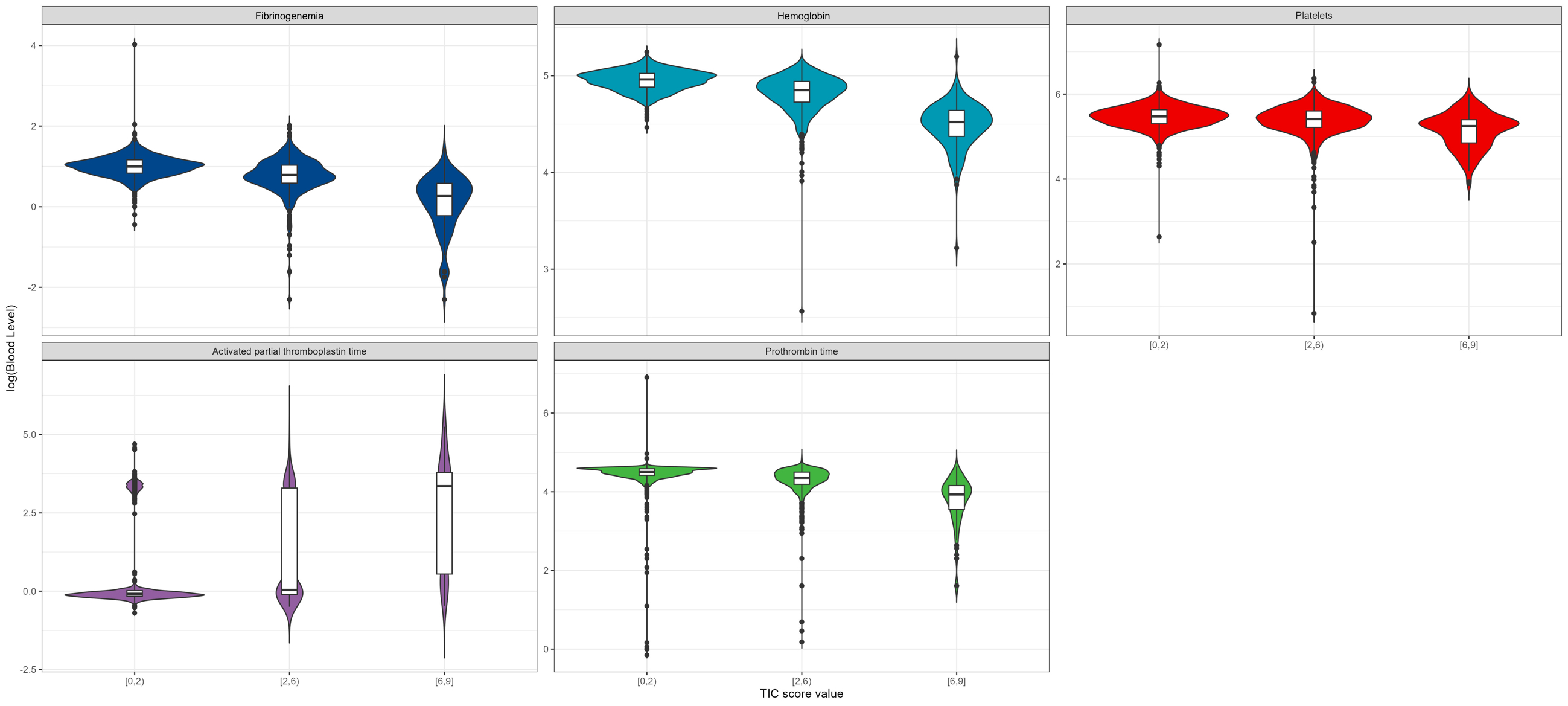

Supplement: Supplementary file 2 — (JPG 453 kb) [file 13054_2024_4955_MOESM2_ESM.jpg]
