# Supplementary material for: Development and validation of the tic score for early detection of traumatic coagulopathy upon hospital admission: a cohort study
Source: Crit Care. 2024 May 18;28:168. doi: 10.1186/s13054-024-04955-7 (PMC11102139; doi:10.1186/s13054-024-04955-7)
Supplement: Supplementary file 3 — (DOCX 12 kb) [file 13054_2024_4955_MOESM3_ESM.docx]

**Supplementary Figure 1.** Hemoglobin, Fibrinogen, PT_ratio_ and platelet count levels (logarithmic scale) according to score category. Violin plot representing the distribution of the log transformation of biomarker levels according to score categories (0 to 1; 2 to 5; and 6 to 9). Boxplots are embedded within the violin plots using white boxes to display median and interquartile range of values. Outliers are indicated by dots.

**Supplementary Figure 2.** Prediction of Fibrinogen < 1.5 g.L^-1^ by TIC Score. ***Panel A***: ROC Curve for the prediction of a fibrinogen < 1.5 g.L^-1^ and a PT_ratio_ > 1.2, by dataset. Blue line indicates Training Set; Sky Blue line indicate Retrospective Set. ***Panel B***: Fibrinogen by score category. Bars correspond to frequency of fibrinogen < 1.5 g.L^-1^. The 95% confidence intervals are displayed for each point using error bars in light grey.
